# Predicting Material Properties of Additively Manufactured Acrylonitrile Butadiene Styrene via a Multiscale Analysis Process

**DOI:** 10.3390/polym14204310

**Published:** 2022-10-13

**Authors:** Phan Quoc Khang Nguyen, Nima Zohdi, Patrick Kamlade, Richard (Chunhui) Yang

**Affiliations:** School of Engineering, Design and Built Environment, Western Sydney University, Penrith, NSW 2751, Australia

**Keywords:** additive manufacturing, acrylonitrile butadiene styrene (ABS), multiscale analysis, finite element method, analytical model, rule of mixtures, material anisotropy

## Abstract

Additive manufacturing (AM) has inherent mechanical strength inconsistencies when the build orientation changes. To address this issue, theoretical models, including analytical and numerical models, can be developed to predict the material properties of additively manufactured materials. This study develops a systematic finite element (FE)-based multiscale numerical model and simulation process for the polymer acrylonitrile butadiene styrene (ABS). ABS samples are fabricated using fused deposition modelling (FDM) to determine the material properties and mechanical behaviours. For macroscale analysis, good agreement between the numerical and experimental tensile strength of transverse samples proved that the FE model is applicable for applying a reverse engineering method in simulating the uniaxial tension of samples. The FE modelling method shows its capability to consider infill density effects. For mesoscale analysis, two methods are developed. The first method is a representative volume element (RVE)-based numerical model for all longitudinal samples. The second method is analytical and based on the rule of mixtures (ROM). Modified rule of mixtures (MROM) models are also developed, which demonstrate an improvement compared to the original ROM models. The research outcomes of this study can facilitate the AM process of parts in various engineering fields.

## 1. Introduction

Additive manufacturing (AM), also known as 3D printing (3DP), rapid prototyping (RP), or solid freedom (SF), is a process of creating 3D model components by gradually joining materials layer by layer. Although it was first introduced three decades ago, it is still gaining a lot of attention from researchers and investors worldwide. Generally, a design can be generated using computer-aided design (CAD) software with complex 3D shapes and exported as a surface tessellation language (STL) file to be compatible with 3D Printer software. Then the slicer software slices the 3D complex model as multiple 2D layers and the resulting toolpath file is sent to the machine for printing. The software also generates a simulation view of the printing process, allowing the designers to verify each printing layer and calculate the estimated time and extruded filaments. The 3DP of polymeric parts prefers thermoplastic polymer materials, such as acrylonitrile butadiene styrene (ABS), polylactic acid (PLA), and polycarbonate (PC), as well as thermosetting polymer materials, including epoxy resins [1].

Since 3DP was first introduced, various 3DP techniques have been developed to accomplish different criteria. Notable current technologies are fused deposition modelling (FDM), powder bed and inkjet head 3DP, stereolithography (SLA), and 3D plotting/direct-write. FDM printing is amongst the most common printing techniques and thermoplastic polymers, such as PC, ABS, and PLA, are often selected as the preferred materials for printing due to their low melting temperatures. Before printing, the quality of the part is controlled in the slicer software by carefully selecting the best setting parameters, such as layer thickness, skirt, support, raster angle, fan speed, printing speed, etc. [1]. In the FDM process, which is the focus of the current study, the filament is fed into a heated liquefier with the assistance of a gearbox. The heat block melts the filaments to a suitable temperature for different thermoplastic materials and feeds them into the nozzle. The nozzle head moves to controlled positions to place molten plastic filaments to form the first layer. After one layer is formed, the nozzle head moves upward and repeats the deposition process to form the next layer [2,3]. Some issues related to FDM printing are void formation during the manufacturing of the parts, layer delamination of the printed polymers, limited choice of thermoplastic polymer materials due to the nature of the method, and material anisotropy [1,4]. Amongst the common issues in FDM, anisotropy of material properties of 3D-printed parts, which can be divided into three categories (mechanical, electrical, and thermal anisotropies), was reported as the most significant issue [5,6,7]. In the studies by Türk et al. and Mohan et al. [8,9], the low-layer adhesion and air voids produced by the printing method were confirmed to be the main reasons for the low mechanical, electrical, and thermal properties of 3D-printed parts printed in transverse build orientation.

According to the review conducted by Zohdi, et al. [6], adding additives [10,11], build orientation [12,13], and bed and nozzle temperatures [14,15] are the top three control factors and parameters that can significantly contribute to mechanical anisotropy. Out of those, the effect of build orientation on the mechanical properties of the printed parts has gained a lot of attention from researchers [6,12,13]. By evaluating the tensile properties of the printed parts, it has been found that the build orientation can affect the mechanical properties of the printed parts and can greatly influence the manufacturing time and the related costs. Layer delamination and air voids from the microstructural analysis were identified as the main reasons for the low strength results and, thus, can be identified as contributing factors in the mechanical anisotropy of printed parts via FDM. In one study conducted by Dul, et al., comparing 3D-printed parts with the compression moulded parts for ABS/Graphene affirmed the effect of build orientation on the mechanical properties of the fabricated samples [16]. In addition to the build orientation influence, material type has an enormous influence on the mechanical properties of the printed parts. In our preliminary study by Zohdi et al., the mechanical anisotropy in parts 3D printed with high-impact polystyrene (HIPS) and ABS was investigated [7]. This study revealed that the samples printed in two different build orientations of longitudinal and transverse with HIPS showed a negligible anisotropy degree compared to ABS samples. SEM images revealed a lower-layer adhesion for the parts made of ABS polymers compared to the HIPS polymers. Moreover, relative tensile strength values of 3D-printed HIPS samples compared to the values for the mould-injected samples prove that by choosing a proper polymer and printing at certain print parameters, almost the same mechanical properties as those of the mould-injected samples could be achieved.

Apart from the mechanical anisotropy, the effects of different printing parameters (infill density, temperature, and layer thickness) on printed parts have also been investigated in literature [17,18,19]. For example, infill density was reported to influence the tensile strength and dynamic mechanical properties of conductive ABS/zinc oxide (CABS/ZnO) composites printed by FDM. Infill density profoundly improved the tensile strength and Young’s modulus properties and decreased the elongation at break. The noticeable increase in dynamic modulus happened when the infill density changed from 50% to 100% [20]. A change in raster angle from 45°/−45° to 90°/0° showed an 8 per cent increase in mechanical strength for ABS [19]. In another study by Rajpurohit, et al. [21], the highest tensile strength was found to be at the 0° raster angle for PLA printed by FDM. The bonding strength was found to vary with the change in build orientation and level of layer thickness. With lower layer thickness, edgewise configuration indicated the highest bonding strength, while with higher layer thickness, flatwise configuration proved to have the highest bonding strength [22]. As stated before, among different types of thermoplastic polymers for 3DP, this article focuses on the parts made of ABS polymer. ABS is a widely used polymer, mostly used in packaging, toys, bottles, housewares, electronic appliances, and light-duty industrial components because of its good rigidity and ease of colouring and processing. However, its applications are limited because of its relatively poor impact strength, heat deflection, and flame retardancy [23].

To compare the experimental results with modelled values, different multiscale modelling methods on CAD programs were referenced by different researchers. For example, sequential multiscale modelling was employed by He, et al. [24] to transfer the effective properties of 3D-braided composites from microscale to mesoscale and from mesoscale to macroscale. One typical method to execute multiscale modelling is constructing a 3D representative volume element (RVE). MSC Digimat-FE software was employed for this method to build 3D microstructure RVE models of aluminium nanocomposites and perform microstructural deformation analysis. The simulation results indicated great agreement between the tensile property of the created RVE models and the experimental values [25]. In another study, MSC Digimat-FE software was also used to construct a 3D microstructural model of BaTiO_3_ to compute the effective elastic constants and epoxy adhesive composites [26].

For extracting the material properties of composites in modelling, the rule of mixtures (ROM), also known as the Voigt model, can be acquired to compute the overall properties based on the properties of each region and its volume fraction [27]. With the analysis of scanning electron microscopy (SEM) images, the volume fraction can be reduced to the area fraction of the cross-section by assuming the constant thickness of testing samples [28,29]. In a study by Deng, et al., the successful application of the ROM to determine the elastic modulus and tensile strength of carbon-fibre-reinforced nylon (CFRN) fabricated by FDM was recorded [28]. Several versions of ROM were employed to validate the experimental results of sisal fibre-reinforced polystyrene composites [27]. For predicting longitudinal properties, Voigt and Halpin–Tsai models showed agreement in the equations. However, the Halpin–Tsai model suggested a correction factor for the transverse properties, while the original Voigt model was modified and replaced by the Reuss model to enhance the accuracy. Applying the developed models to derive the properties of the samples printed in longitudinal build orientation exhibited good agreement with experimental results, while higher discrepancies were observed for transverse properties, especially for lower fibre loadings [27].

In this study, a systematic multiscale modelling and simulation process is developed to model the 3D-printed parts made of ABS polymer at the macro- and mesolength scales, respectively. Firstly, the macroscale analysis is devised using FE modelling as the reverse engineering method and it is employed to investigate the effects of infill density at different build orientations. Secondly, the mesoscale analysis is developed by devising two methods to extract the material properties of the ABS polymers. The first RVE-based numerical method investigates the effect of the process parameters, while the second Rule-of-Mixtures-based analytical method evaluates differences in material properties at two build orientations and aims to highlight the effect of mechanical anisotropy. Lastly, the numerical and analytical results were compared with the experimental results and a modified rule of mixtures was proposed. The whole paper is outlined in the following sections: Section 2 focuses on the development of the experimental and numerical analysis procedure; Section 3 presents the obtained results and conducts the discussion; and Section 4 draws conclusions and outlooks for future work.

## 2. Experimental and Numerical Analysis

### 2.1. Sample Design and Experimental Procedure

Polymer pellets of ABS were used to extrude filaments with an outer diameter of 1.75 ± 0.07 mm. Pellets of ABS polymer grade PA747 (C_8_H_8_·C_4_H_6_·C_3_H_3_N)_n_ were purchased from the ChiMei Corporation, Taiwan. The dogbone samples were designed according to the ASTM D638 standard [30]. The standard geometry and dimensions of selected Type V were employed to create the CAD model of the dogbone samples using SolidWorks.

**Figure 1:**
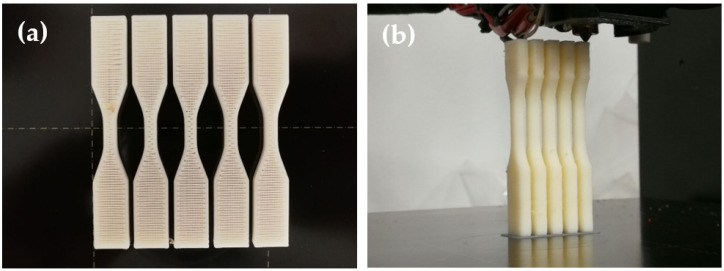
ABS samples in printing: (**a**) one batch of five longitudinal samples; and (**b**) one batch of five transverse samples.

Samples were fabricated in batches of five with a Prusa i3 MK3S+ printer and its embedded slicer (Figure 1). Five replications for each parameter were prepared to minimise the error percentage. Four sets of samples were produced to investigate the effects of different printing parameters, including infill density, layer thickness, raster angle, and temperature. For each printing parameter, the samples were fabricated at two different build orientations: longitudinal and transverse. For the infill density set, other machine factors were set as default, while infill density was modified to 40%, 60%, 80%, and 100%, respectively. Next, the layer thickness set was created with the same method by changing the layer thickness to 0.15 mm, 0.25 mm, and 0.35 mm, respectively. Next, samples in the raster angle set were produced at ±45° to compare with the original 0°/90° raster angle. Finally, the printing temperature was varied from 220 °C to 280 °C with an increment of 20 °C to complete the temperature set.

An Instron 3365 machine with a 5-kN load cell was employed to extract the mechanical properties of produced samples. From the raw data of the five samples from each batch, the average ultimate tensile strength and Young’s modulus were calculated as a benchmark for the simulation process.

SEM images were collected using a Hitachi Flex SEM 1000 II machine. The fractured surfaces of the dogbone samples from mechanical testings were flattened and prepared with the assistance of a microtome instrument prior to imaging. Samples were first submerged into liquid nitrogen for at least 20 min and then immediately prepared using a microtome instrument. Images were collected at various magnification levels to fit the purpose of modelling.

### 2.2. Macroscale Analysis

**Figure 2:**
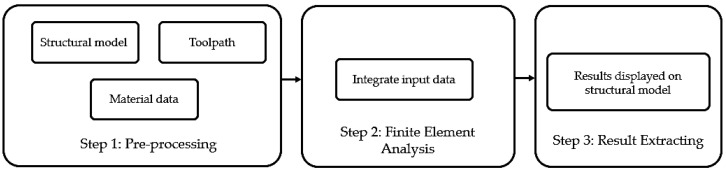
FEA workflow for macroscale modelling.

The macroscale model and analysis were developed for the tensile specimens to perform static structural analysis using ANSYS Workbench and MSC Digimat Software. Its three-step workflow is displayed in Figure 2.

The CAD model of the tensile specimens was created using SolidWorks and then imported into ANSYS Workbench to devise a standard static structural analysis with appropriate boundary conditions, according to the ASTM D638 standard. Meanwhile, the toolpath containing moving tools’ locations in 3D space was generated using the Prusa slicer and the material data were prepared with the mean-field theory using MSC Digimat-MF. Next, all those data were further used as inputs in the FE model developed using MSC Digimat-RP to simulate the 3DP process of the tensile specimens. ANSYS Solver was used to perform finite element analysis (FEA) via the interface between MSC Digimat and ANSYS. Finally, the results were viewed and displayed on the structural model in ANSYS Workbench for final data processing to extract the macroscopic failure indicator (MFI) values and then tensile strength.

Since the parts printed with ABS polymer had transverse isotropic behaviours, the material’s failure was modelled using the Tsai–Hill criteria available in MSC Digimat. The Tsai–Hill criteria require the input parameters as axial tensile strength (X), in-plane tensile strength (Y), and transverse shear strength (S) to assign an MFI value to each element. Those parameters can be extracted from the properties of samples printed at 0/0 raster angle–longitudinal build orientation and 90/90 raster angle–transverse build orientation. However, most currently available printers, including the employed printers for this project, could not accomplish the necessary settings for the above raster angles. Hence, the macroscale analysis was used to apply the reverse engineering method to extract the required parameters and match the tensile strength obtained for the sample manufactured at 100% infill density. The tensile strength was the maximum equivalent von-Mises stress when the MFI values in a location of the specimen exceeded 1.0. For calibration purposes that required multiple trials, this process was conducted at an element size of 1 mm.

In FEA, the result accuracy is strongly influenced by the element size. With a smaller element size, more elements are generated, thus, improving the accuracy of the results. Nonetheless, it also increases the computation time and might exceed the hardware limitation. Therefore, a mesh convergence test was conducted to derive the appropriate element size, guaranteeing accuracy and saving computation time for the macroscale analysis. For the convergence test, the maximum equivalent von-Mises stress was selected as the scrutinised criterion.

In 3DP, the printing time can be reduced at lower infill densities, but the 3D-printed materials experience more air voids. Air void is a crucial defect affecting the material’s mechanical properties and the software must recognise the infill density effect to successfully simulate the properties of 3D-printed parts [6,7,31]. Therefore, the final aim of the macroscale analysis was to investigate the software’s capabilities in detecting the infill density effect. The toolpath files that contained the infill density information were replaced accordingly for each infill density ranging from 40% to 100% with an increment of 10%.

### 2.3. Mesoscale Analysis

The process of mesoscale modelling adopted the SEM images to create the representative volume elements (RVEs) and perform the FE analysis.

**Figure 3:**
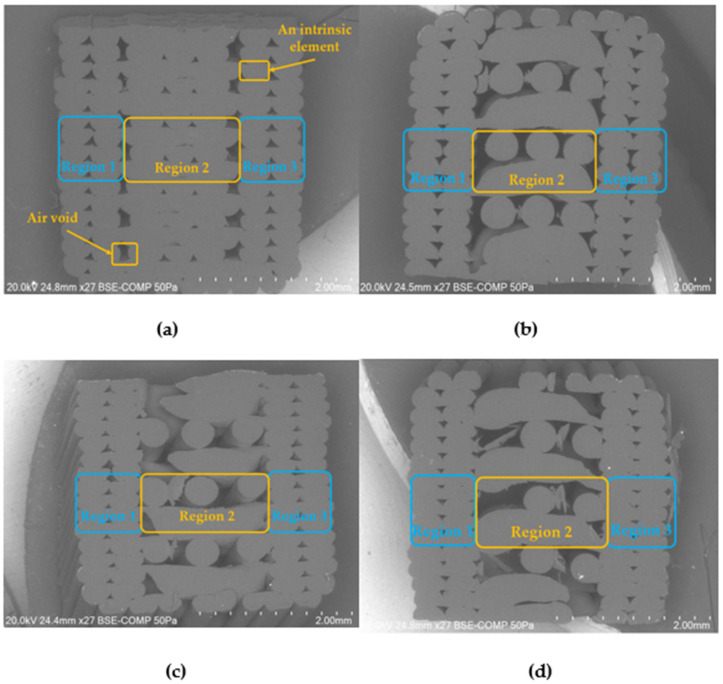
SEM image of ABS at (**a**) 100%, (**b**) 80%, (**c**) 60%, (**d**) and 40% infill density and longitudinal orientation.

Figure 3 shows the SEM image of ABS-fabricated samples printed with 100%, 80%, 60%, and 40% infill density and longitudinal orientation, respectively. In these images, Regions 1 and 3, framed in blue, were identified as perimeter regions, while Region 2 represents the infill, which is framed in yellow. With the information from the SEM images, two methods were developed to study the material properties (tensile strength) of 3D-printed samples in MSC Digimat-FE. The results can be generalised to compare with experimental data by assuming the ultimate tensile strength occurs in the middle (gauge length) region of the dogbone specimens.

**Figure 4:**
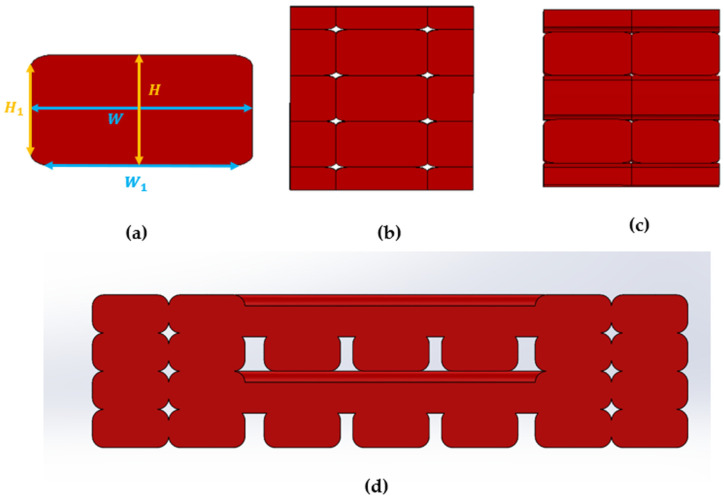
(**a**) Intrinsic element; (**b**) Regions 1 and 3—aligned model; (**c**) Region 2—sparse model; and (**d**) full RVE model.

From the SEM image, one typical intrinsic element in each region was selected to measure the maximum width (W), maximum height (H), edge’s width (W_1_), and edge’s height (H_1_) to define the shape of the intrinsic element depicted in Figure 4a. In the first method, the CAD model of the entire structure as shown in Figure 4d, including perimeter and infill regions, was designed and analysed using MSC Digimat FE. The second method utilised the printing parameters, including nozzle diameter (0.4 mm) and layer thickness (0.2 mm), and manipulated the available models, the aligned and sparse models, to replicate the perimeter and infill regions as shown in Figure 4b and Figure 4c, respectively. After that, the tensile strength of the entire structure was computed using ROM, as shown in Equation (1). In this equation, $X_{P},\;X_{I,}\;X_{\mathrm{Total}}$ are the tensile strength of the perimeter region, infill region, and the resultant model combining all three regions, respectively. By selecting the constant thickness for all regions, the volume fraction $V_{P}$ $\left(0\leq V_{P}\leq 1\right)$ of the perimeter region to the resultant model was reduced to only define area fraction from the SEM images [28].

(1) $$X_{P}V_{P}+\left(1-V_{p}\right)X_{I}=X_{\mathrm{Total}}\;\left(\mathrm{Voigt}\;\mathrm{Model}\right)$$

With the same limitations described for the macroscale analysis in mind, the original material data for all methods were pre-calibrated to match the mechanical properties of the samples printed at 100% of infill density in the first method. Then the same material file was used to perform simulations for other sets to maintain consistency. Then, in each set (for instance: infill density, layer thickness, etc.), the properties of longitudinal and transverse orientations were extracted by modifying the loading directions onto the RVE models.

The first method was capable of generating the results for all four sets of parameters, including infill density, layer thickness, and temperature of the ABS polymer, when the selected build orientation was set as longitudinal. The FE model was automatically meshed with the non-conforming tetra method with local mesh refinements. The generated stress–strain curve at the macroscopic level was recorded to extract the mechanical properties. For the second method, a mesh convergence test was conducted, and an appropriate element size of 0.025 mm was applied to generate data for the infill density at both longitudinal and transverse orientations.

To the best of the author’s knowledge, no studies previously reported the use of ROM for the same purpose in the second method. Hence, the original ROM can be modified to enhance its accuracy. Based on the original ROM and the obtained data, an additional term was included to adjust the ROM suitably for each percentage of the infill density. Equations (2) and (3) were used previously in other studies, mostly focusing on composite and nanocomposite materials [32]. However, so far, these equations were not used to describe the issues associated with the ROM for the polymer parts printed with AM. In these equations, additional functions of f(x) and g(x) were inserted into the original ROM to predict the material properties of the longitudinal and the transverse samples, respectively. In both additional functions, the variable x is the percentage of infill density. For instance, if the sample is fabricated at 80% infill density, the value of x is 0.8. For this study, the evaluated function was set in a quartic form, a polynomial of degree four with the same form as P(x) in Equation (4), and the coefficients of the functions $a_{0},{\;a}_{1},{\;a}_{2},{\;a}_{3},{\;a}_{4}$ were derived in MATLAB using the collected data. The outcome of this task was expected to assist future modellings of the samples fabricated at different infill densities with the same pure polymer and enhance the accuracy of the original model.

(2) $$X_{P}V_{P}+f\left(x\right)\left(1-V_{p}\right)X_{I}=X_{\mathrm{Total}}\left(\mathrm{MROM}\;\mathrm{for}\;\mathrm{the}\;\mathrm{longitudinal}\right)$$

(3) $$X_{P}V_{P}+g\left(x\right)\left(1-V_{p}\right)X_{I}=X_{\mathrm{Total}}\left(\mathrm{MROM}\;\mathrm{for}\;\mathrm{the}\;\mathrm{transverse}\right)$$

(4) $$P\left(x\right)=a_{4}x^{4}+a_{3}x^{3}+a_{2}x^{2}+a_{1}x+a_{0}$$

## 3. Results and Discussion

### 3.1. Experimental Results

Table 1 provides the data to illustrate the effects of infill density, layer thickness, raster angle, and nozzle temperature on the tensile strength of the 3D-printed ABS samples, respectively. Further, the associated root square error percentages (error mean) for the tensile strength are included in the tables.

According to Table 1, the tensile strength of the ABS at both build orientations increases when the infill density increases from 40% to 100% and the 100% infill density offers the best values. As can be seen from the results, samples printed in longitudinal build orientation can experience an increase of around 33% and around 65% for the ones printed in transverse build orientation when the infill percentage increases from 40% to 100%. In a critical review conducted by Syrlybayev et al. [33], the same behaviour was found in numerous studies where increasing the infill percentage can increase mechanical tensile strength. The tensile strength of ABS-fabricated samples is traditionally between 28 MPa and 120 MPa and the 3D-printed ABS with 100% infill density fabricated with the longitudinal orientation in the current study has values falling into this range comparably. It is worth mentioning that the discrepancies between the longitudinal and transverse build orientations in 3D-printed parts correspond to the anisotropy phenomena. When the load is applied parallel to the build orientation, the printed filaments of polymers resist the applied force, but when the load is perpendicular to the direction of the printing, parameters, such as air voids and layer-adhesion quality, come into effect before the structural chain of the polymer is changed. To minimise these discrepancies, optimising the printing conditions can lead to improved mechanical properties of the 3D-printed parts, regardless of the orientation of the applied force or the build orientation.

The effects of layer thickness on the tensile strength of the 3D-printed ABS can be also found in Table 1. It can be seen that a thickness of 0.25 mm provides the highest value of the tensile strength by 15% when compared with 0.15 layer thickness and 6.6% when compared with the 0.35-mm layer thickness for the samples printed in longitudinal build orientation. However, the samples printed in transverse build orientation with the 0.15-mm layer thickness demonstrate the best tensile strengths value by 28.6% and 16% when compared with those of 0.35 mm and 0.25 mm, respectively. Similar behaviour of the effect of layer thickness was also observed in a study conducted by Kuznetsov et al. [34]. Further, the samples printed with the 0.15-mm layer thickness showed the minimum amount of anisotropy between the samples printed in longitudinal build orientation and transverse build orientation.

The machine settings limit the setting of the raster angle and only two raster angles can be set as 0°/90° and ±45°. Effects of raster angle on the tensile strength of the 3D-printed ABS can be also found in Table 1. The 0°/90° raster angle provides around 20.6% higher value for samples printed in longitudinal build orientation and around 43.6% higher values for the samples printed in transverse build orientation. These findings are in line with those found by Odell et al. [35]. Further, the samples printed with 0°/90° raster angle showed around a 17% less discrepancy between the samples printed in longitudinal build orientation and transverse build orientation and, therefore, less anisotropy behaviour can be observed for these samples.

Lastly, the effects of varying the nozzle temperature from 220 °C up to 280 °C can be found in Table 1. It can be seen that a nozzle temperature of 260 °C offers the highest value of the tensile strength for the 3D-printed ABS samples printed in longitudinal build orientation, while a 220 °C nozzle temperature provides the lowest value. Similarly, the highest value for the samples printed in transverse build orientation was recorded for the samples printed at 260 °C, but the lowest value was recorded for the samples printed at 280 °C. It is also worth mentioning that a similar trend in mechanical properties by increasing the nozzle temperature was observed in a study conducted by Aliheidari et al. [36]. Therefore, anisotropy was recorded to be the lowest for samples printed at 260 °C.

### 3.2. Macroscale Analysis

#### 3.2.1. Development of the Reverse Engineering Method

**Figure 5:**
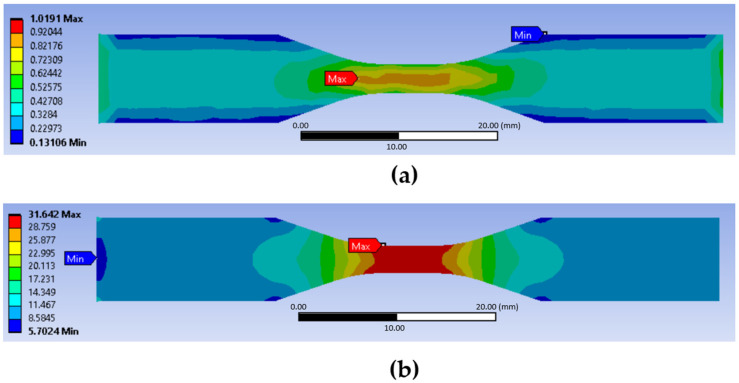
(**a**) Macroscopic failure indicator (MFI); and (**b**) equivalent stress (longitudinal orientation).

Figure 5a,b represent the MFI and equivalent stress distributions for the sample built at the longitudinal orientation. It is worth mentioning that the MFI in Figure 5a is dimensional with no units and the von-Mises stress values in Figure 5b are in MPa. With the maximum MFI and equivalent stress values displayed in the middle of the dogbone sample, the tensile strength was confirmed to occur in this region.

Table 2 compares the experimental and simulation results. The simulation results were calibrated for both build orientations and close relations between the simulation and experimental results were observed.

#### 3.2.2. Mesh Convergence Test for Macroscale Structure

Table 3 represents the results of the mesh convergence test at various mesh sizes. When the maximum MFI values first exceeded 1, the maximum equivalent stress at that time step was recorded in Table 3. The resulting element type was SOLID186 and one node has 3 degrees of freedom (DOFs). As the mesh size decreased, the running time significantly increased proportionally with the total number of nodes. According to the outcomes of the mesh sensitivity analysis, a mesh size smaller than 0.30 mm was proved suitable for considering toolpath information at each printing layer. For a macroscale structure and to ensure accuracy, the mesh size of 0.25 mm was adopted in the current study.

#### 3.2.3. Effects of the Infill Density on Material Performance

In this study, the effects of the infill density are presented using void volume fraction, defined as SVAR3 in the output file of static structural analysis available in ANSYS. The obtained results are shown in Table 4. In the numerical simulations, the local infill content was set per element; thus, average void volume fractions throughout the samples were recorded.

**Figure 6:**
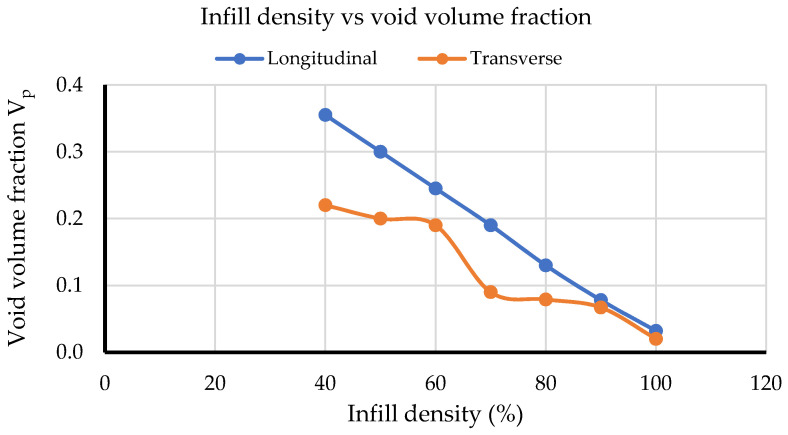
Infill density vs. void volume fraction.

Figure 6 illustrates the change in void volume fraction at different infill densities for both build orientations. From the results, only the curve for longitudinal orientation is observed to approximate a linear line, although both curves successfully show higher void volume fraction at lower infill densities. Therefore, for the macroscale structure, modelling software successfully differentiated the effect of infill densities for longitudinal and transverse orientations.

### 3.3. Mesoscale Analysis

#### 3.3.1. Numerical Results of Infill Density, Temperature, Raster Angle, and Layer Thickness (Method 1)

**Figure 7:**
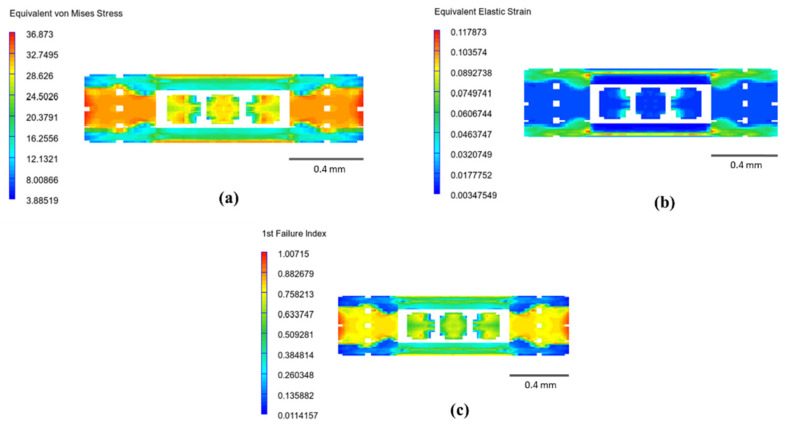
Infill density of 80% at longitudinal orientation: (**a**) equivalent von-Mises stress; (**b**) equivalent elastic strain; and (**c**) 1st failure index.

Figure 7 shows the stress, strain, and failure index distributions for the model with 80% of infill density and longitudinal orientation. With force applied perpendicular to the viewing plane, the highest stress is observed at the perimeter region, indicating an outstanding contribution of this region to the overall strength. The result also explains why the elements in the perimeter region have the highest failure index and appear to approach failure mode earlier than other elements. It is worth pointing out that the von-Mises stress values in Figure 7a are in MPa, while values in Figure 7b,c are dimensionless.

**Figure 8:**
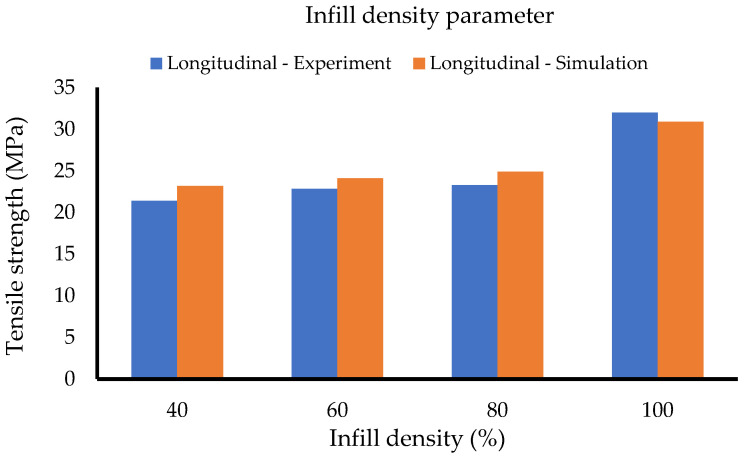
Infill density set (Method 1).

Table 5 and Figure 8 summarise the obtained simulation results for the infill density set for the first method. From the graph, the obtained tensile strengths for the longitudinal orientation samples succeed at showing a close relation and the same trend as the experimental data.

**Figure 9:**
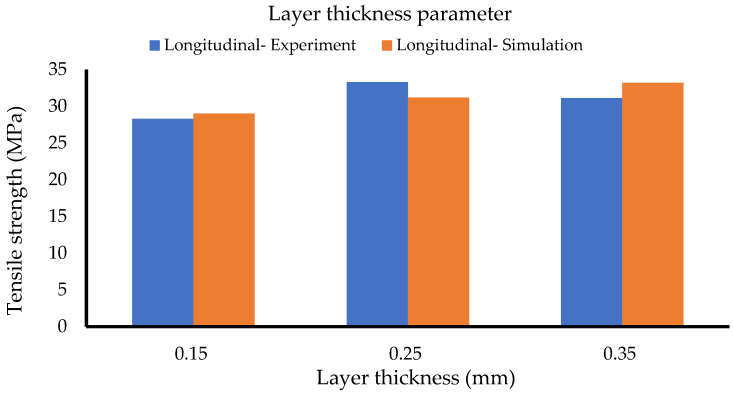
Layer thickness set (Method 1).

Table 6 and Figure 9 illustrate the results for the layer thickness batch of samples. While the longitudinal experimental data indicate a peak at the 0.25-mm thickness, the simulation data have a peak at the 0.35-mm thickness and an increasing trend. It is essential to note that the structures for 0.25-mm and 0.35-mm from the SEM images exhibited defections and unexpected air voids and gaps.

**Figure 10:**
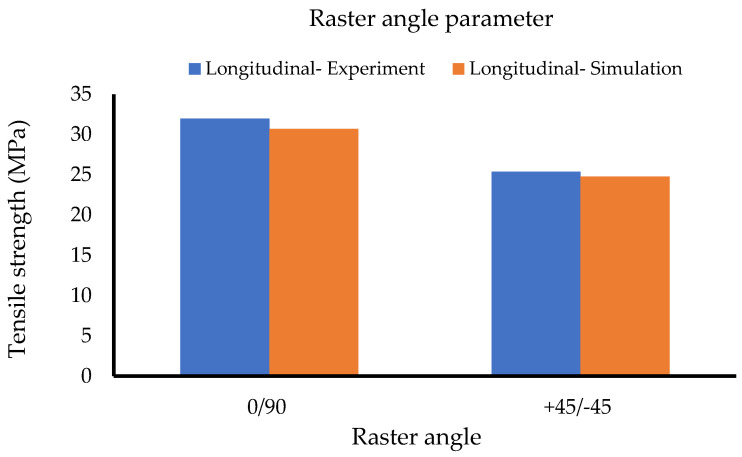
Raster angle set (Method 1).

Table 7 and Figure 10 represent the collected data for the raster angle set. The graph illustrates similar simulation and experimental results. The simulation results also classify the use of a 0/90 raster angle in the practical 3DP process for generating higher tensile strengths.

**Figure 11:**
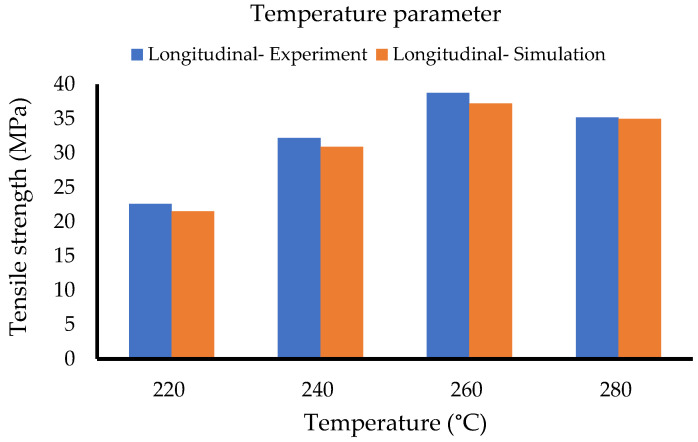
Temperature set (Method 1).

The collected data for the temperature set are summarised in Table 8 and Figure 11. The simulation data for the longitudinal samples indicate a high accuracy and the same trend as the experimental data.

In summary, the simulation results for all four printing sets showed high accuracies for the longitudinal models. The accuracies of the numerical simulations can be further enhanced by obtaining the SEM images of the two remaining 3D views to reproduce the RVE structures entirely. Further, a more reliable method, such as image processing, can replace manual measurement of the intrinsic elements’ dimensions from the SEM images. With the main advantage of generating the full RVE model containing both the perimeter and the infill regions, the first method proves applicable for validating the results from the experiments.

#### 3.3.2. Numerical Results of the Infill Density Set (Method 2)

**Figure 12:**
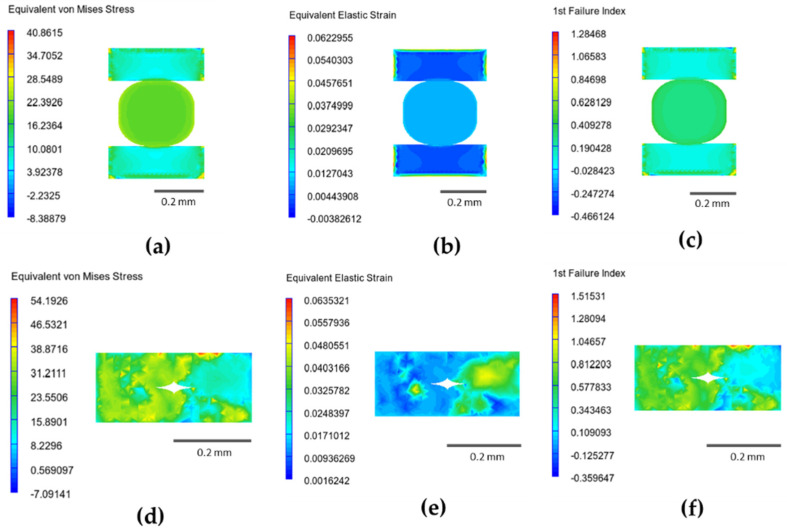
Infill density of 80% at longitudinal orientation: (**a**) equivalent von-Mises stress—infill region; (**b**) equivalent elastic strain—infill region; (**c**) 1st failure index—infill region; (**d**) equivalent von-Mises stress—perimeter region; (**e**) equivalent elastic strain—perimeter region; and (**f**) 1st failure index—perimeter region.

Figure 12 comprises the stress, strain, and first failure index distribution graphs for the perimeter and infill regions of the 80% infill density at longitudinal orientation. With the applied force perpendicular to the viewing plane, more significant distributions of stress, strain, and the first failure index are observed for the infill region, while the perimeter region shows high distributions on the left area. For both regions, the higher stress area results in a higher first failure index. It should be highlighted that the von-Mises stress values in Figure 12a,d are in MPa, while values in Figure 12b–f have no units.

**Figure 13:**
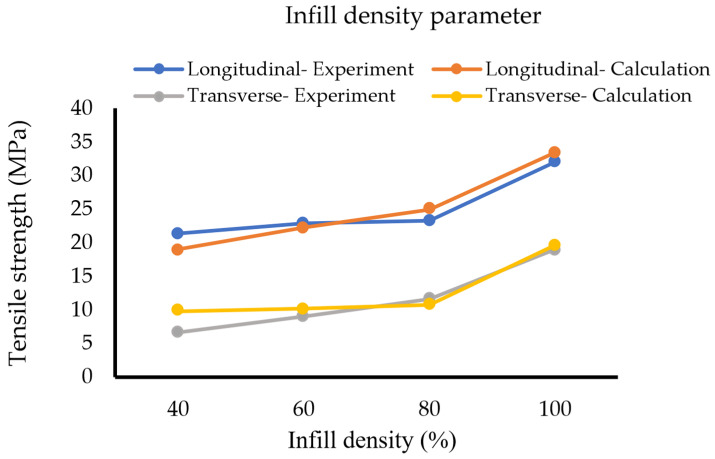
Infill density set (Method 2).

Table 9 and Table 10 and Figure 13 represent the results for the infill density set using the second method. With the same material data, the results of the calculations show good agreement with the experiment for both build orientations, especially for 80% and 100% of infill densities. The calculated data succeed at illustrating the mechanical anisotropy effect by showing the different properties of the longitudinal and the transverse parts. From the table, the tensile strength of the infill region is significantly lower than the perimeter region at lower infill densities and with the volume fraction of the perimeter as 0.5, the perimeter confirms to contribute 50% to the overall tensile strength. However, significant deviations are observed at the lower infill densities. The reason is that with no negative factors in the formula and a fixed tensile strength of the perimeter, the ROM ultimately results in a flattening curve, even though the infill region keeps decreasing. These flattening behaviours are evidenced in Figure 13 at 80%, 60%, and 40% of the transversely oriented samples, respectively.

It is worth mentioning that the modelled intrinsic element’s shapes (Figure 4a) and the volume fraction of the perimeter region for the second method were purely determined by the provided printing information of the nozzle diameter of 0.4 mm and the geometrical dimensions of the dogbone samples. Thus, if the ROM formula can be modified to increase the efficiency at the lower infill densities, the second method can create a new opportunity to predict the mechanical properties without needing the SEM images, which only capture the cross-section after the tensile testing. In other words, with the known dimensions of the structure from the ASTM standard, the properties of the 3D-printed samples can be computed by applying this method accordingly for each cross-section and, finally, extracting the overall properties. However, since the second method employs the default models from the software and assumes the 3D-printed structure does not consist of unexpected defections, it requires more effort to model the unpredicted behaviours observed in other printing parameter sets (temperature, layer thickness, and raster angle). Consequently, this method was only applied to the infill density set in this study.

#### 3.3.3. Development of the Modified Rule of Mixtures (MROM)

**Figure 14:**
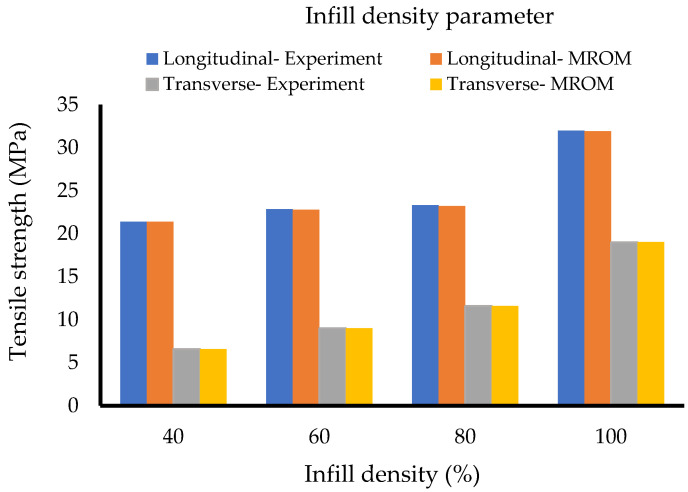
Infill density set (MROM).

Table 11 represents the derived coefficients for the additional functions, while Figure 14 displays the calculated tensile strength after applying MROM. From the charts, the additional functions succeed at matching the calculated results with the experimental. Furthermore, results in Section 3.2.3 also confirm that different ROM models must be developed for evaluating the properties of the longitudinal and transverse samples. The proposed MROM will be employed in future projects to validate the material properties of the same ABS polymer samples printed at different infill densities, such as 50%, 70%, and 90%, and compare its efficiency with the original ROM.

## 4. Conclusions

In this study, the systematic FE-based multiscale numerical modelling and simulation processes, including the FE-based model at the macroscale and two methods at the mesoscale, were successfully developed for the ABS polymer samples printed using the FDM method.

For the proposed macroscale analysis, the following conclusions can be drawn:

- With the 2.7% difference between the numerical and experimental tensile strength of transverse samples, the macroscale analysis revealed that the reverse engineering method can be used to determine material data, considering the effects of build orientation and infill density, which provides a close fit with those experimental data for the longitudinally built samples.
- The longitudinal build orientation exhibits linear properties related to the change in the infill density, while the transverse build orientation does not show any specific trends due to its unpredicted behaviours.

At the mesoscale, the mesoscale analysis of the polymeric samples extracted the following research findings:

- With the process of reproducing the entire RVE structure, including the perimeter and infill regions, the tensile strength results using the first method showed close relations with experimental data of longitudinally built samples.
- Further, with less than 10% error percentages, the first method was proved to be the best to replicate those unexpected behaviours, considering printing parameter sets, e.g., layer thickness, raster angle, and temperature.
- By employing ROM, the calculated tensile strengths using the second method have close relations with those experimental data at higher infill densities and significant deviations at lower infill densities.
- For the transversely built samples, the modified model of ROM was proposed using parameters to improve the prediction accuracy.

Future work can be attempted to reproduce more accurate intrinsic models by obtaining all the SEM images of the cross-sections’ front, right, and top views and, thus, will wholly demonstrate the natural defections caused by the 3DP process. Furthermore, the devised macroscale and mesoscale modelling and analysis procedures can also be employed to model the nanocomposites’ material properties.

## Figures and Tables

**Table 1:** Effects of infill density, layer thickness, raster angle and nozzle temperature on tensile strength.

	Tensile Strength (MPa)			
Infill Density (%)	Longitudinal	Error (Mean)	Transverse	Error (Mean)
40	21.4	0.35	6.6	0.43
60	22.9	0.19	9	0.4
80	23.3	0.47	11.6	0.4
100	32	0.71	19	0.61
**Layer Thickness (mm)**	**Longitudinal**	**Error (Mean)**	**Transverse**	**Error (Mean)**
0.15	28.3	1.13	17.1	0.49
0.25	33.3	0.45	12.2	0.19
0.35	31.1	0.19	14.3	0.36
**Raster Angle**	**Longitudinal**	**Error (Mean)**	**Transverse**	**Error (Mean)**
0°/90°	32	0.71	19	0.61
± 45°	25.4	0.27	10.7	0.43
**Nozzle Temperature (°C)**	**Longitudinal**	**Error (Mean)**	**Transverse**	**Error (Mean)**
220	22.6	0.27	9	0.51
240	32.4	1.36	9.7	0.62
260	38.8	0.34	19	0.61
280	35.2	0.22	7.9	0.13

**Table 2:** Outcomes using the reverse engineering method.

Results	ABS 100% Tensile Strength (MPa)	
	Longitudinal	Transverse
**Experimental**	32.0	19.0
**Simulation**	31.6	18.5
**Error (%)**	1.2%	2.7%

**Table 3:** Mesh convergence test for macroscale structure.

Maximum Element Size (mm)	Total Number of Nodes	Total DOFs	Maximum Equivalent (Von-Mises) Stress (MPa)	Running Time with 1 CPU (min)
1.00	10,862	32,586	31.6	3
0.75	22,043	66,129	28.6	4
0.50	64,349	193,047	27.9	15
0.40	111,691	335,073	22.9	21
0.30	248,583	745,749	21.2	50
0.25	451,158	1,353,474	21.5	92

**Table 4:** Void volume fraction at various infill densities.

Infill Density (%)	Void Volume Fraction	
	Longitudinal	Transverse
100	0.032	0.020
90	0.078	0.067
80	0.130	0.079
70	0.190	0.090
60	0.245	0.190
50	0.300	0.200
40	0.355	0.220

**Table 5:** Modelling results for the infill density set (Method 1).

Infill Density (%)	Tensile Strength (MPa)		
	Longitudinal		
	Experiment	Simulation	Error (%)
40	21.4	23.2	8.4
60	22.9	24.1	5.4
80	23.3	24.9	6.9
100	32.0	30.9	3.4

**Table 6:** Modelling results for the layer thickness set (Method 1).

Layer Thickness (mm)	Tensile Strength (MPa)		
	Longitudinal		
	Experiment (MPa)	Simulation (MPa)	Error (%)
0.15	28.3	29.0	2.5
0.25	33.3	31.2	6.3
0.35	31.1	33.2	6.8

**Table 7:** Modelling results for the raster angle set (Method 1).

Raster Angle	Tensile Strength (MPa)		
	Longitudinal		
	Experiment (MPa)	Simulation (MPa)	Error (%)
0°/90°	32.0	30.7	4.1
±45°	25.4	24.8	2.4

**Table 8:** Modelling results for the temperature set (Method 1).

Temperature (°C)	Tensile Strength (MPa)		
	Longitudinal		
	Experiment (MPa)	Simulation (MPa)	Error (%)
220	22.6	21.5	4.9
240	32.2	30.9	4.0
260	38.8	37.2	4.0
280	35.2	35.0	0.6

**Table 9:** Modelling results for the infill density set—longitudinal orientation (Method 2).

Infill (%)	Tensile Strength-Perimeter Region (MPa)	Tensile Strength-Infill Region (MPa)	Volume Fraction of Perimeter Region	Calculated Tensile Strength (MPa)	Experimental Tensile Strength (MPa)	Error (%)
40	36.3	1.7	0.5	19.0	21.4	11.3
60	36.3	8.2	0.5	22.3	22.9	2.7
80	36.3	13.6	0.5	25.0	23.3	7.1
100	36.3	30.6	0.5	33.5	32.0	4.5

**Table 10:** Modelling results for the infill density set—transverse orientation (Method 2).

Infill (%)	Tensile Strength-Perimeter Region (MPa)	Tensile Strength-Infill Region (MPa)	Volume Fraction of Perimeter Region	Calculated Tensile Strength (MPa)	Experimental Tensile Strength (MPa)	Error (%)
40	18.9	0.7	0.5	9.8	6.6	48.5
60	18.9	1.5	0.5	10.2	9.0	13.6
80	18.9	2.6	0.5	10.7	11.6	7.4
100	18.9	20.4	0.5	19.7	19.0	3.4

**Table 11:** Coefficients of the additional functions for MROM.

	$a_{4}$	$a_{3}$	$a_{2}$	$a_{1}$	$a_{0}$
f(x)	−33.88	57.58	0	−39.70	16.90
g(x)	54.07	−102.94	0	87.91	−38.10

## Data Availability

The data presented in this study are available upon request from the corresponding author.
